# Two-stage designs optimal under the alternative hypothesis for phase II cancer clinical trials

**DOI:** 10.1016/j.cct.2010.07.008

**Published:** 2010-11

**Authors:** A.P. Mander, S.G. Thompson

**Affiliations:** a MRC Biostatistics Unit Hub for Trials Methodology Research, Institute of Public Health, Robinson Way, Cambridge, CB2 0SR, United Kingdom; b MRC Biostatistics Unit, Institute of Public Health, Robinson Way, Cambridge, CB2 0SR, United Kingdom

**Keywords:** Two-stage designs, Optimal design, Simon's two-stage design, Phase II trials

## Abstract

The Simon two-stage optimal design is often used for phase II cancer clinical trials. A study proceeds to the second stage unless the null hypothesis, that the true tumour response rate is below some specified value, is already accepted at the end of stage one. The conventional optimal design, for given type 1 and type 2 error rates, is the one which minimises the expected sample size under the null hypothesis. However, at least some new agents are active, and designs that explicitly address this possibility should be considered. We therefore investigate novel designs which are optimal under the alternative hypothesis, that the tumour response rate is higher than the null hypothesis value, and also designs which allow early stopping for efficacy. We make available, software for identifying the corresponding optimal and minimax designs. Considerable savings in expected sample sizes can be achieved if the alternative hypothesis is in fact true, without sample sizes suffering too much if the null hypothesis is true. We present an example discussing the merits of different designs in a practical context. We conclude that it is relevant to consider optimal designs under a range of hypotheses about the true response rate, and that allowing early stopping for efficacy is always advantageous in terms of expected sample size.

## 1 Introduction

The main aim of phase II cancer clinical trials is to evaluate the anti-tumour effect of a treatment, screening out agents that are insufficiently active and selecting active agents for future studies [1]. Efficacy is often determined by whether a treatment causes a tumour response (or not) in a small single arm trial [2] and interest is primarily in making the decision to progress an agent to a randomised phase III comparative study. The evidence to make this decision is evaluated by testing the null hypothesis that the true response rate is less than or equal to some pre-specified value. It is desirable to achieve this goal whilst minimising the number of patients exposed to a novel agent and this led to the development of single arm designs that allowed stopping for efficacy or futility. The Fleming design [3] did this by calculating critical values for accepting and rejecting the null hypothesis using the O'Brien and Fleming multiple testing procedure [4]. This design allowed early stopping but only controlled type 1 and type 2 errors and there was no attempt to be “optimal” in terms of minimising the expected sample size; this was introduced by the Simon two-stage design [5].

The Simon two-stage design only considers stopping for futility and the optimal design has the smallest expected sample size when the null hypothesis is true. This paper investigates a novel definition of optimality based on the expected sample size when the alternative hypothesis, that the response rate is greater than the pre-specified value, is true. The rationale for only stopping for futility is that many novel agents do not work, and we want to minimise the number of patients exposed to an inactive drug, but in reality there are sufficient active agents to consider also stopping for efficacy [2]. One of the justifications given for only allowing stopping for futility in the Simon design was that when an agent has substantial activity there is interest in studying additional patients in order to estimate the proportion, extent and durability of response [5]. However, stopping for efficacy may save drug development time, bring useful treatments into clinical practice quicker, and should reduce costs [6].

In a recent review of study designs in cancer, over 20*%* of all phase II studies with a reported statistical design were Simon designs and 45*%* were two-stage designs [2]. The main reasons for stopping trials early were futility (69*%*) and efficacy (13*%*); other reasons were toxicity and poor accrual. Many of the designs reviewed did not include stopping for efficacy as a possibility and the studies would have benefitted, in terms of expected sample size, had they been optimal.

There have been several extensions to the Simon two-stage design including the optimal three-stage design [7], optimal three-stage design stopping for efficacy [6], admissible designs that balance the optimisation criteria of expected sample size and maximum sample size [8], using a predictive probability design [9], balanced two-stage designs [10], and estimating response rates after using the Simon two-stage design [11]. All of these papers only consider the optimal design under the null hypothesis. This paper explores optimal two-stage designs, with stopping for futility and efficacy, under the alternative hypothesis. These new designs retain the same hypotheses and initial specification of the type 1 and type 2 errors as the Simon two-stage design. We consider only single arm studies, not randomised phase II trials.

## 2 Method

### 2.1 Simon two-stage design

The hypotheses about the true response rate (*p*) to be tested by this single arm design are,

$$\begin{array}{l} H_{0}:p\leq p_{0},\\ H_{1}:p=p_{1}>p_{0}\text{,} \end{array}$$

where *p*_0_ is the pre-specified fixed null response probability and *p*_1_ is the minimum desired response probability required to progress the treatment to a later stage trial. Typical values for *p*_0_ are below 0.3 and for the target improvement rate (*p*_1_ − *p*_0_) are between 0.1 and 0.2 [2].

Each Simon two-stage design [5] is indexed by four numbers, *r*_1_, *r*, *n*_1_ and *n*. The study is stopped early for futility if there are ≤ *r*_1_ responders out of *n*_1_ participants at stage 1, and the null hypothesis is not rejected. Otherwise the study proceeds to the second stage, with a total sample size *n*, and the null hypothesis is not rejected if there ≤ *r* responders at the end of the study. It is referred to as an “*r*_1_ / *n*_1_ *r* / *n*” design. Values for *r*_1_, *r*, *n*_1_ and *n* are found for fixed *p*_0_, *p*_1_, *α* (the type 1 error probability) and *β* (the type 2 error probability). A type 1 error occurs when there are > *r*_1_ responders at the end of stage 1 and > *r* responders at the end of the study when *p* = *p*_0_. A type 2 error occurs if there are ≤ *r*_1_ responders in stage 1 or ≤ *r* responders at the end of the study when *p* = *p*_1_.

The probability of not rejecting the null hypothesis, $\bar{R}(p)$, is a function of the true response rate *p*. The number of responders *X*, based on a true response rate *p* and sample size *m*, has a Binomial distribution, and we write the distribution functions as *P*(*X* = *x*) = *b*(*m*, *p*, *x*) and *P*(*X* ≤ *x*) = *B*(*m*, *p*, *x*). It follows that the probability $\bar{R}(p)$ is as follows[5]:

(1) $$\bar{R}(p)=B(n_{1},p,r_{1})+\sum_{i=r_{1}+1}^{min(n_{1},r)}b(n_{1},p,i)B(n-n_{1},p,r-i)\text{.}$$

An acceptable design is one that satisfies the error probability constraints $\bar{R}(p_{0})\geq 1-\alpha$ and $\bar{R}(p_{1})\leq \beta$ and let Ω be the set of all such designs. A grid search is used to go through every combination of *r*_1_, *r*, *n*_1_ and *n* with an upper limit for *n*, usually between 0.85 and 1.5 times the sample size for a single stage design [12]. The probability of terminating the study early, *PET(p)*, is the first term in Eq. (1), *B*(*n*_1_, *p*, *r*_1_), and the expected sample size for this design, *E*(*N*|*p*), is *n*_1_*PET*(*p*) + *n*(1 − *PET*(*p*)). The optimal design under *H*_0_ is the one in Ω that has the smallest expected sample size *E*(*N*|*p*_0_) and the minimax design has the smallest *E*(*N*|*p*_0_) amongst those designs in Ω with the smallest *n*. This paper will refer to the former as the *H*_0_-optimal and the latter as the *H*_0_-minimax design; these are the traditional Simon two-stage designs [5].

Before investigating stopping for efficacy, we consider the optimal and minimax designs when minimising *E*(*N*|*p*_1_) but keeping the same type 1 and type 2 errors. These designs will be labelled the *H*_1_-optimal and *H*_1_-minimax designs, respectively. The probability of not rejecting the null hypothesis is the same as Eq. (1) but with the argument *p* = *p*_1_. When the true response is *p*_1_ then the probability of early termination for futility must be smaller than the type 2 error, as seen below in Eq. (2), and so the expected sample size will be greater than *βn*_1_ + (1 − *β*)*n*. For *β* near 0 the expected sample size is close to the upper bound of *n*, and the optimal design will be the one with the smallest *n*. So for studies with high power (small *β*) the *H*_1_-optimal and *H*_1_-minimax designs will be very similar.

(2) $$\begin{array}{l} \text{By definition},\bar{R}(p_{1})\leq \beta \;\text{for an acceptable design}.\\ \text{But}\;B(n_{1},p_{1},r_{1})\leq \bar{R}(p_{1})\text{,}\;\text{from equation 1}\\ \text{So PET}(p_{1})\leq \beta \text{.} \end{array}$$

### 2.2 Stopping for efficacy

It is possible to reduce expected sample sizes by terminating early for efficacy as well as futility. One obvious design is to stop for efficacy at the first stage when there are greater than *r* responders, since the criterion to reject the null hypothesis at the end of the second stage has already been met. Although this would have little efficiency advantage under *H*_0_ [5] because there is only a small chance of stopping for efficacy, this will not necessarily be true for designs that are optimal under *H*_1_.

We consider here a less conservative design that allows for early stopping for efficacy when there are > *r*_2_ responders at the first stage. The full design is as follows:

• recruit *n*_1_ participants,
  – stop for futility if there are ≤ *r*_1_ responders,
  – stop for efficacy if there are > *r*_2_ responders (where *r*_2_ ≤ *r*, and *r*_1_ < *r*_2_ ≤ *n*_1_),
• recruit a further *n*_2_ participants (where *n* = *n*_1_ + *n*_2_),
  – Do not reject *H*_0_ if there are ≤ *r* responders,
  – Reject *H*_0_ if there are > *r* responders.

This design is now indexed by 5 numbers and will be referred to as a “(*r*_1_ *r*_2_) / *n*_1_ *r* / *n*” design.

Using the same notation as previously, using suffix *E* to represent stopping for efficacy or futility, the probability of not rejecting the null hypothesis is as follows:

$${\bar{R}}_{E}(p)=B(n_{1},p,r_{1})+\sum_{i=r_{1}+1}^{r_{2}}b(n_{1},p,i)B(n-n_{1},p,r-i)\text{.}$$

This equation only differs from Eq. (1) in that the summation upper limit is now bounded by *r*_2_. As before we require that the error probabilities are controlled by ${\bar{R}}_{E}(p_{0})\geq 1-\alpha$ and ${\bar{R}}_{E}(p_{1})\leq \beta$. The probability of early termination for this design is as follows:

$$PET_{E}(p)=1-B(n_{1},p,r_{2})+B(n_{1},p,r_{1})\text{.}$$

For the same values of *r*_1_ and *n*_1_, *PET*_*E*_(*p*) ≥ *PET*(*p*). The optimal and minimax designs are found using a grid search given an upper limit for *n*, and for *p*_0_ or *p*_1_, and these designs are labelled *H*_0_-optimalE, *H*_0_-minimaxE, *H*_1_-optimalE and *H*_1_-minimaxE.

### 2.3 Implementation

A program has been written to find the minimax and optimal designs under either *H*_0_ or *H*_1_ with stopping for efficacy and futility, or with stopping only for futility, using Stata 11 [13]. This command is freely available and can be downloaded via Stata using the command *ssc install simon2stage*. The program uses the Mata language to do a grid search of all the possible values of *r*, *r*_1_, *r*_2_, *n*_1_ and *n*, has options to select stopping only for futility, or stopping for futility and efficacy, and can find the optimal or minimax designs for any value of the true response probability *p*. Only results for *p* = *p*_0_ and *p* = *p*_1_ are used in this paper.

## 3 Results

### 3.1 Optimal designs

Optimal designs were found for a set of design parameters *p*_0_, *p*_1_, *α* and *β* that were first used in the Simon two-stage design paper [5], which included a comparison to single stage designs, and then in subsequent papers [6,7,14]. Our designs are shown for *p*_1_ − *p*_0_ = 0.2 for *p*_0_ = 0.05, 0.1 and 0.3 and (*α*, *β*) = (0.1, 0.1), (0.05, 0.2) and (0.05, 0.1). These target values were within the range of typical null response rates and target improvement rates reported in phase II cancer studies [2].

Table 1 displays the minimax and optimal designs under *H*_0_ and *H*_1_ with and without efficacy stopping for *p*_0_ = 0.05 and *p*_1_ = 0.25. In this case the minimax designs are the same when considering the null or alternative response probabilities. Stopping for efficacy always gives smaller expected sample sizes. Stopping for efficacy gives the largest gain in efficiency for the *H*_0_-minimax design when (*α*, *β*) = (0.05, 0.1): the expected sample size under *H*_0_ improves from 20.4 to 18.5 and under *H*_1_ from 24.9 to 16.7. Stopping for efficacy has a much larger impact on *E*(*N*|*p*_1_) than *E*(*N*|*p*_0_) because *PET*(*p*_1_) is small for designs which do not allow stopping for efficacy and large for those that do.

The design with the smallest *E*(*N*|*p*_1_) = 13.0 is the *H*_1_-optimalE design for (*α*, *β*) = (0.1, 0.1) and is indexed as (0 1) /10 3/26; the study continues to a second stage only if there is a single event in the first 10 participants. If the true treatment effect is *p*_1_ then the probability of stopping for efficacy is 0.812. In fact this design also has the smallest value for *E*(*N*|*p*_0_) + *E*(*N*|*p*_1_) in Table 1 for (*α*, *β*) = (0.1, 0.1) and so performs well under both hypotheses. The corresponding *H*_1_-optimal design has an expected sample size of *E*(*N*|*p*_1_) = 19.8 so the gains can be substantial if the treatment is efficacious. The traditional Simon two-stage design, the *H*_0_-optimal design, has the highest expected sample size *E*(*N*|*p*_1_) = 22.9 compared to all the other designs.

Table 2 displays the minimax and optimal designs under *H*_0_ and *H*_1_ with and without efficacy stopping for *p*_0_ = 0.1 and *p*_1_ = 0.3. The minimax designs now show some considerable differences under *H*_0_ and *H*_1_: the first stages are different for the minimax designs not stopping for efficacy but the second stages *r* / *n* are the same. There is not a clear consistency in the direction of the change in *n*_1_, it is 16 for the *H*_0_-minimax design and 11 for *H*_1_-minimax design when (*α*, *β*) = (0.1, 0.1). In contrast to this, for the other type 1 and 2 errors, *n*_1_ is bigger under *H*_1_ than under *H*_0_. However the impact on the expected sample sizes is small. Stopping for efficacy in minimax designs usually results in a reduction in expected sample sizes but this is not the case when (*α*, *β*) = (0.05, 0.2) where *E*(*N*|*p*_0_) increases. The *H*_0_-optimal design has a smaller *E*(*N*|*p*_0_) than all the minimax designs but has a much higher value (up to over 50*%*) for *E*(*N*|*p*_1_). In Table 2 the *H*_1_-optimal designs are exactly the same as the *H*_1_-minimax designs. The *H*_0_-optimalE and *H*_1_-optimalE designs give small expected sample sizes and have similar values for *E*(*N*|*p*_0_) + *E*(*N*|*p*_1_) but the *H*_1_-optimalE design has much smaller values of *n*. So optimising under *H*_1_ also seems to control the maximum sample size.

Table 3 displays the minimax and optimal designs under *H*_0_ and *H*_1_ with and without efficacy stopping for *p*_0_ = 0.3. The *H*_0_-optimal designs have much larger expected sample sizes *E*(*N*|*p*_1_) than the *H*_0_-minimax design. Stopping for efficacy lowers *E*(*N*|*p*_1_) substantially but the effects on *E*(*N*|*p*_0_) differ depending on the error probabilities. For (*α*, *β*) = (0.1, 0.1) the expected sample size *E*(*N*|*p*_0_) was 35.0 for the *H*_0_-minimax design but was 32.7 for the *H*_0_-minimaxE design. However when (*α*, *β*) = (0.05, 0.2) it rose from 25.7 to 30.7 because *n*_1_ increased from 19 to 27 and *n* decreased from 39 to 36. The *H*_1_-optimal designs are again exactly the same as the *H*_1_-minimax designs. Stopping for efficacy can also slightly increase *n* as seen for the *H*_0_-optimal design with (*α*, *β*) = (0.1, 0.1). Amongst the optimal designs the *H*_1_ designs have smaller second stages than the *H*_0_ designs and bigger first stages, but this does not have a huge impact on *PET*(*p*_1_). Again the *H*_1_-optimalE design has the smallest values for *E*(*N*|*p*_1_) + *E*(*N*|*p*_0_) except for (*α*, *β*) = (0.1, 0.1) where the *H*_0_-optimalE design is best on this metric.

### 3.2 Acceptable designs for a range of *n*

**Fig. 1:**
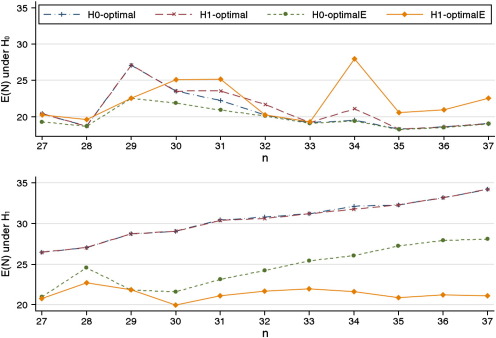
Expected sample sizes given (*p*_0_, *p*_1_, *α*, *β*) = (0.1, 0.3, 0.05, 0.15) for different values of the maximum sample size *n*.

Both the *H*_0_-minimax and the *H*_0_-optimal designs have been widely applied in the literature and other designs have been largely ignored. However, these two designs can give highly divergent characteristics. For example the *H*_0_-minimax can lead to a much smaller maximum sample size than the *H*_0_-optimal design [8]; given the design parameters (*p*_0_, *p*_1_, *α*, *β*) = (0.1, 0.3, 0.05, 0.15), then the *H*_0_-minimax design is 2/18 5/27 and the *H*_0_-optimal design is 1/11 6/35. Using the same design parameters Fig. 1 shows the expected sample sizes under *H*_0_ and *H*_1_ for each optimal design over a range of values for *n* starting from the maximum sample size for the *H*_0_-minimax design. The figure shows that the *H*_0_-optimal and *H*_1_-optimal designs give almost identical expected sample sizes under *H*_1_ and these increase approximately linearly with *n*. Under *H*_1_, only the *H*_1_-optimalE design does not have a generally increasing expected sample size. Under *H*_0_, the *H*_0_-optimalE designs always have the smallest expected sample sizes and the *H*_1_-optimalE designs have some larger expected sample sizes but the difference is variable.

## 4 Application of the *H*_1_-optimalE design

A recent paper studying the effects of Pazopanib on soft tissue sarcoma (STS) [15] reported the results from four Simon two-stage designs; the Simon two-stage designs were used independently in four different strata defined by STS type. Each design assumed (*p*_0_, *p*_1_, *α*, *β*) = (0.2, 0.4, 0.1, 0.1) and the *H*_0_-optimal design was 3/17 10/37 with *E*(*N*|*p*_0_) = 26.0, but *E*(*N*|*p*_1_) = 36.1. At the end of the study the observed response probabilities were, in order of magnitude, 5/19, 16/41, 18/41 and 18/37. The first stratum had 3 responses and stopped after the first stage although subsequently 2 responses were re-classified into this stratum. The middle two strata recruited 4 extra patients beyond the originally planned sample size. Assuming the observed responses reflected the true response rates then the probabilities of stopping for futility were 0.31, 0.06, 0.02 and 0.01 and the resulting expected sample sizes were 30.8, 35.9, 36.5 and 36.8; it is clear that the studies were unlikely to stop for futility. With the same design parameters the *H*_1_-optimalE design is (1 5) /15 11/38; assuming the same true response rates the probabilities of stopping early are 0.24, 0.57, 0.71 and 0.82 and the expected sample sizes are 32.4, 24.8, 21.6 and 19.1. By adding the expected sample sizes over the four strata, the total expected sample size for the *H*_0_-optimal design is 140 and for the *H*_1_-optimalE design is 97.9; on average this new design would have required 42 fewer patients. A single stage design would have required a sample size of 36 per stratum with the null hypothesis being rejected if there were ≥ 11 responders. In totalthe study would have required 144 patients, slightly more than the *H*_0_-optimal design.

## 5 Discussion

This article highlights that if researchers have an agent that is effective then the Simon two-stage design is not an optimal design. In the extreme case, if the true response probability is 1, the study will never stop for futility and the expected sample size is *n*. In the less extreme case, for a true response probability of *p*_1_, there is a chance smaller than *β* of stopping the study; so studies with high power have only a small chance of being stopped early for futility. All the *H*_1_-optimal designs reduce the expected sample size under the alternative compared to the traditional designs. However, this comes at the cost of slightly larger expected sample sizes under the null. The main effect of stopping for efficacy is that the probabilities of early termination increase, under both hypotheses, leading to efficiency gains over a broader set of true response probabilities.

In designing studies there is a choice whether to minimise the expected sample size or to minimise the maximum sample size. Our results show that the minimax design is beneficial if a treatment is efficacious: for this case the *H*_1_-optimal and *H*_0_-minimax designs were very similar. The only differences between the *H*_1_-optimal and *H*_0_-minimax designs were in the first stage; optimising under *H*_1_ and not stopping for efficacy has a small probability of early termination when *p* = *p*_1_ and hence the size of *n* dominates the expected sample size. It is clear from the results presented (Tables 1–3) that the best designs vary between situations with different design parameters; this is because of the discrete nature of the binomial distribution and exact probability calculations involved.

Only a few papers have investigated designs optimal for alternative values of the true response. Simon [16] explored a few values that were close to *p*_0_ in designs that only stopped for futility and did not minimise the expected sample size, Shuster [17] looked at minimising the maximum expected sample size over a range of response rates and Hanfelt et al. [18] considered minimising the median sample size. Our designs allow stopping for efficacy and focus on minimising the expected sample size under the alternative hypothesis. Another paper proposed an adaptive version of the Simon two-stage design [12]. This design considers two alternative response rates, *p*_1_ and *p*_2_, and the size of the second stage depends on the number of responders in the first stage. One optimality criterion was to minimise the maximum of expected sample sizes at the null and two response rates, $max\left\{E(N|p_{i}),i=0,1,2\right\}$. The design developed here differs in that it is specified before the study begins but can consider a range of possible true response probabilities. It is still reliant on the null and alternative values for the true probability of response. Another approach would be to elicit a prior distribution for the true response probability and find a design that minimises the expected sample size over this distribution. This is likely to be difficult in practice because the discreteness of the Binomial distribution leads to a lack of ordering of designs and might require a discrete prior to be computationally possible. The other effect of the discreteness is that a single type of design is not uniformly more efficient than any other and the best design depends on what the true response probability is.

Newer treatments, such as cytostatic drugs may require randomised phase II studies [19] and these studies can also incorporate an interim analysis to stop for efficacy or futility. Any two-stage randomised design should consider optimality criteria with both the alternative and null values as possible true response rates. The effect of changing the optimality criteria in extensions of the Simon single arm two-stage design, such as the three-stage design, could also be assessed. It would also be possible to use other definitions of optimality such as minimising *E*(*N*|*p*_0_) + *E*(*N*|*p*_1_). The methods here could be further improved by using a curtailed sampling approach [14] and stopping each part as soon as the desired number of responders has been reached. If the aim of the study is to estimate the response probability then the equivalence between p-values and confidence intervals can be used to obtain interval estimates [11,20].

This article has presented a new set of designs that consider stopping for efficacy as well as futility under *H*_0_ and *H*_1_. All the designs can be found using freely available software implemented in the common statistical package Stata. Although our focus has been on tumour response in cancer trials, these designs could be used for any binary outcome. The issues covered here should stimulate more discussion during the design of new studies and encourage consideration of a broader range of possibilities instead of using traditional designs to fit all situations.

## Figures and Tables

**Table 1:** Minimax and optimal designs, with and without stopping for efficacy and under both hypotheses when *p*_0_ = 0.05 and *p*_1_ = 0.25.

(*α*, *β*)	Design			*E*(*N*|*p*_0_)	*E*(*N*|*p*_1_)	*PET*(*p*_0_)	*PET*(*p*_1_)
(0.1,0.1)	*H*_0_-minimax	0/13	2/20	16.4	19.8	0.513	0.024
(0.05,0.2)	0/12	2/16	13.8	15.9	0.540	0.032
(0.05,0.1)	0/15	3/25	20.4	24.9	0.463	0.013
(0.1,0.1)	*H*_1_-minimax	0/13	2/20	16.4	19.8	0.513	0.024
(0.05,0.2)	0/12	2/16	13.8	15.9	0.540	0.032
(0.05,0.1)	0/15	3/25	20.4	24.9	0.463	0.013
(0.1,0.1)	*H*_0_-minimaxE	(0 2)/13	2/20	16.2	15.2	0.538	0.691
(0.05,0.2)	(0 2)/12	2/16	13.8	13.4	0.560	0.641
(0.05,0.1)	(0 2)/13	3/25	18.5	16.7	0.538	0.691
(0.1,0.1)	*H*_1_-minimaxE	(0 2)/13	2/20	16.2	15.2	0.538	0.691
(0.05,0.2)	(0 2)/12	2/16	13.8	13.4	0.560	0.641
(0.05,0.1)	(0 2)/13	3/25	18.5	16.7	0.538	0.691
(0.1,0.1)	*H*_0_-optimal	0/9	2/24	14.5	22.9	0.630	0.075
(0.05,0.2)	0/9	2/17	12.0	16.4	0.630	0.075
(0.05,0.1)	0/9	3/30	16.8	28.4	0.630	0.075
(0.1,0.1)	*H*_1_-optimal	0/13	2/20	16.4	19.8	0.513	0.024
(0.05,0.2)	0/12	2/16	13.8	15.9	0.540	0.032
(0.05,0.1)	0/15	3/25	20.4	24.9	0.463	0.013
(0.1,0.1)	*H*_0_-optimalE	(0 2)/9	2/24	14.4	16.9	0.639	0.474
(0.05,0.2)	(0 2)/9	2/17	11.9	13.2	0.639	0.474
(0.05,0.1)	(0 3)/9	3/30	16.8	24.9	0.631	0.241
(0.1,0.1)	*H*_1_-optimalE	(0 1)/10	3/26	15.0	13.0	0.685	0.812
(0.05,0.2)	(0 2)/9	2/17	11.9	13.2	0.639	0.474
(0.05,0.1)	(0 2)/13	3/25	18.5	16.7	0.538	0.691

**Table 2:** Minimax and optimal designs, with and without stopping for efficacy and under both hypotheses when *p*_0_ = 0.1 and *p*_1_ = 0.3.

(*α*, *β*)	Design			*E*(*N*|*p*_0_)	*E*(*N*|*p*_1_)	*PET*(*p*_0_)	*PET*(*p*_1_)
(0.1,0.1)	*H*_0_-minimax	1/16	4/25	20.4	24.8	0.515	0.026
(0.05,0.2)	1/15	5/25	19.5	24.6	0.549	0.035
(0.05,0.1)	2/22	6/33	26.2	32.8	0.620	0.021
(0.1,0.1)	*H*_1_-minimax	0/11	4/25	20.6	24.7	0.314	0.020
(0.05,0.2)	2/18	5/25	19.9	24.6	0.734	0.060
(0.05,0.1)	3/25	6/33	26.9	32.7	0.764	0.033
(0.1,0.1)	*H*_0_-minimaxE	(1 4)/16	4/25	20.2	19.8	0.532	0.576
(0.05,0.2)	(2 4)/19	5/24	20.3	20.2	0.741	0.764
(0.05,0.1)	(1 4)/16	6/33	24.0	23.2	0.532	0.576
(0.1,0.1)	*H*_1_-minimaxE	(0 3)/11	4/25	20.3	18.7	0.332	0.450
(0.05,0.2)	(0 3)/13	5/24	20.8	17.5	0.288	0.589
(0.05,0.1)	(1 4)/16	6/33	24.0	23.2	0.532	0.576
(0.1,0.1)	*H*_0_-optimal	1/12	5/35	19.8	33.0	0.659	0.085
(0.05,0.2)	1/10	5/29	15.0	26.2	0.736	0.149
(0.05,0.1)	2/18	6/35	22.5	34.0	0.734	0.060
(0.1,0.1)	*H*_1_-optimal	0/11	4/25	20.6	24.7	0.314	0.020
(0.05,0.2)	2/18	5/25	19.9	24.6	0.734	0.060
(0.05,0.1)	3/25	6/33	26.9	32.7	0.764	0.033
(0.1,0.1)	*H*_0_-optimalE	(1 3)/13	5/31	19.2	19.4	0.656	0.643
(0.05,0.2)	(1 4)/10	5/29	15.0	23.3	0.738	0.300
(0.05,0.1)	(2 4)/17	7/41	22.2	24.5	0.784	0.689
(0.1,0.1)	*H*_1_-optimalE	(0 2)/9	5/30	20.8	17.9	0.440	0.578
(0.05,0.2)	(0 3)/13	5/24	20.8	17.5	0.288	0.589
(0.05,0.1)	(1 4)/16	6/33	24.0	23.2	0.532	0.576

**Table 3:** Minimax and optimal designs, with and without stopping for efficacy and under both hypotheses when *p*_0_ = 0.3 and *p*_1_ = 0.5.

(*α*, *β*)	Design			*E*(*N*|*p*_0_)	*E*(*N*|*p*_1_)	*PET*(*p*_0_)	*PET*(*p*_1_)
(0.1, 0.1)	*H*_0_-minimax	7/28	15/39	35.0	38.9	0.365	0.006
(0.05, 0.2)	6/19	16/39	25.7	37.3	0.666	0.084
(0.05, 0.1)	7/24	21/53	36.6	52.1	0.565	0.032
(0.1, 0.1)	*H*_1_-minimax	10/33	15/39	35.4	38.9	0.599	0.018
(0.05, 0.2)	7/21	16/39	26.0	37.3	0.723	0.095
(0.05, 0.1)	7/24	21/53	36.6	52.1	0.565	0.032
(0.1, 0.1)	*H*_0_-minimaxE	(7 12)/26	15/39	32.7	31.3	0.486	0.592
(0.05, 0.2)	(8 13)/27	15/36	30.7	31.3	0.592	0.526
(0.05, 0.1)	(11 17)/37	20/50	42.5	41.7	0.579	0.639
(0.1, 0.1)	*H*_1_-minimaxE	(5 11)/23	15/39	34.4	30.9	0.290	0.505
(0.05, 0.2)	(6 12)/24	15/36	31.2	30.8	0.400	0.431
(0.05, 0.1)	(7 15)/31	20/50	45.2	40.5	0.254	0.502
(0.1, 0.1)	*H*_0_-optimal	7/22	17/46	29.9	44.4	0.671	0.067
(0.05, 0.2)	5/15	18/46	23.6	41.3	0.722	0.151
(0.05, 0.1)	8/24	24/63	34.7	60.0	0.725	0.076
(0.1, 0.1)	*H*_1_-optimal	10/33	15/39	35.4	38.9	0.599	0.018
(0.05, 0.2)	7/21	16/39	26.0	37.3	0.723	0.095
(0.05, 0.1)	7/24	21/53	36.6	52.1	0.565	0.032
(0.1, 0.1)	*H*_0_-optimalE	(6 9)/20	18/47	29.3	29.6	0.656	0.646
(0.05, 0.2)	(5 11)/15	18/46	23.6	40.8	0.722	0.168
(0.05, 0.1)	(8 14)/24	24/63	34.7	54.0	0.726	0.230
(0.1, 0.1)	*H*_1_-optimalE	(5 9)/21	18/45	34.7	28.6	0.430	0.681
(0.05, 0.2)	(4 9)/18	16/38	30.9	29.5	0.354	0.423
(0.05, 0.1)	(7 11)/24	24/59	38.1	37.6	0.596	0.613
